# c-Maf regulates pluripotency genes, proliferation/self-renewal, and lineage commitment in ROS-mediated senescence of human mesenchymal stem cells

**DOI:** 10.18632/oncotarget.6178

**Published:** 2015-10-19

**Authors:** Pei-Min Chen, Chia-Hua Lin, Nan-Ting Li, Yao-Ming Wu, Ming-Tsan Lin, Shih-Chieh Hung, Men-Luh Yen

**Affiliations:** ^1^ Department of Obstetrics/Gynecology, College of Medicine, National Taiwan University, Taipei, Taiwan; ^2^ Research Center for Developmental Biology and Regenerative Medicine, National Taiwan University, Taipei, Taiwan; ^3^ Department of Surgery, National Taiwan University Hospital and National Taiwan University College of Medicine, Taipei, Taiwan; ^4^ Department of Medical Education and Bioethics, Graduate Institute of Medical Education and Bioethics, College of Medicine, National Taiwan University, Taipei, Taiwan; ^5^ Institute of Clinical Medicine, National Yang-Ming University, Taipei, Taiwan; ^6^ Department of Medical Research, Taipei Veterans General Hospital, Taipei, Taiwan

**Keywords:** human adipose tissue-derived MSCs (hAMSCs), ROS-mediated senescence, c-Maf, self-renewal, lineage commitment, Gerotarget

## Abstract

Mesenchymal stem cells (MSCs) are therapeutically relevant multilineage and immunomodulatory progenitors. Ex vivo expansion of these rare cells is necessary for clinical application and can result in detrimental senescent effects, with mechanisms still largely unknown. We found that vigorous *ex vivo* expansion of human adipose tissue-derived MSCs (hAMSCs) results in proliferative decline, cell cycle arrest, and altered differentiation capacity. This senescent phenotype was associated with reactive oxygen species (ROS) accumulation, and with increased expression of G1 cell -cycle inhibitors— p15^INK4b^ and p16^INK4a^ — but decreased expression of pluripotency genes—Oct-4, Sox-2, Nanog, and c-Myc—as well as c-Maf a co-factor of MSC lineage-specific transcription factor and sensitive to oxidative stress. These global changes in the transcriptional and functional programs of proliferation, differentiation, and self-renewal were all mediated by ROS-induced suppression of c-Maf, as evidenced by binding of c-Maf to promoter regions of multiple relevant genes in hAMSCs which could be reduced by exogenous ROS. Our findings implicate the strong effects of ROS on multiple stem cell functions with a central role for c-Maf in stem cell senescence.

## INTRODUCTION

Human adipose tissue-derived mesenchymal stem cells (hAMSCs) represent an adult multipotent stem cell population with the capacity to differentiate into the mesodermal lineages of adipocytes, osteoblasts, and chondrocytes [1]. This source of tissue-specific MSCs are especially advantageous for therapeutic application, because they can be easily obtained, and lack the ethical issues surrounding other stem cell sources [2]. Like most adult stem cell sources, hAMSCs require ex vivo expansion to reach the high cell volumes required for clinical use; this has been reported to induce cellular senescence in adult bone marrow (BM) MSCs in which proliferative capacity is decreased and potential of genetic and epigenetic alterations is increased [3, 4]. In somatic cells, senescence can be defined as a stable arrest of the cell cycle coupled to stereotyped phenotypic changes [5]. Additionally with stem cells, senescence appears to affect differentiation capacity as well, but mechanism are just beginning to be revealed [6] and lineage-specific changes have not been demonstrated consistently [7-9].

As cells undergo senescence, oxidative stress and the resultant elevation of reactive oxygen species (ROS) including hydrogen peroxide (H_2_O_2_) are known to be critical factors involved in the process [10]. In post-mitotic somatic cells, a rise in the level of ROS can damage various cell components and activate specific signaling pathways to trigger distinct cellular programs including cell cycle arrest and low metabolic activity to arrive at a senescent phenotype [11]. However, direct and detailed mechanisms regarding how these senescence-related cellular programs and phenotypes are mediated are just beginning to be elucidated. Moreover, adult stem cells have a distinct biological role and niche compared to somatic cells, and effects of senescence and mechanisms involved are likely different to that found with post-mitotic somatic cells. Since adipose tissue is increasingly popular as a source for MSCs, we studied the process of senescence and mechanisms involved in these somatic progenitors. We found that senescence of hAMSCs was associated with increased ROS, which affected both proliferative and differentiation capacity. ROS alters these two major cellular programs in hAMSCs via c-Maf, a transcription factor (TF) [12], to affect differentiation capacity as well as exert direct transcriptional control on proliferation-related and pluripotency genes.

## RESULTS

### hAMSCs express BMMSC markers and have tri-mesodermal differentiation capability

**Figure 1:**
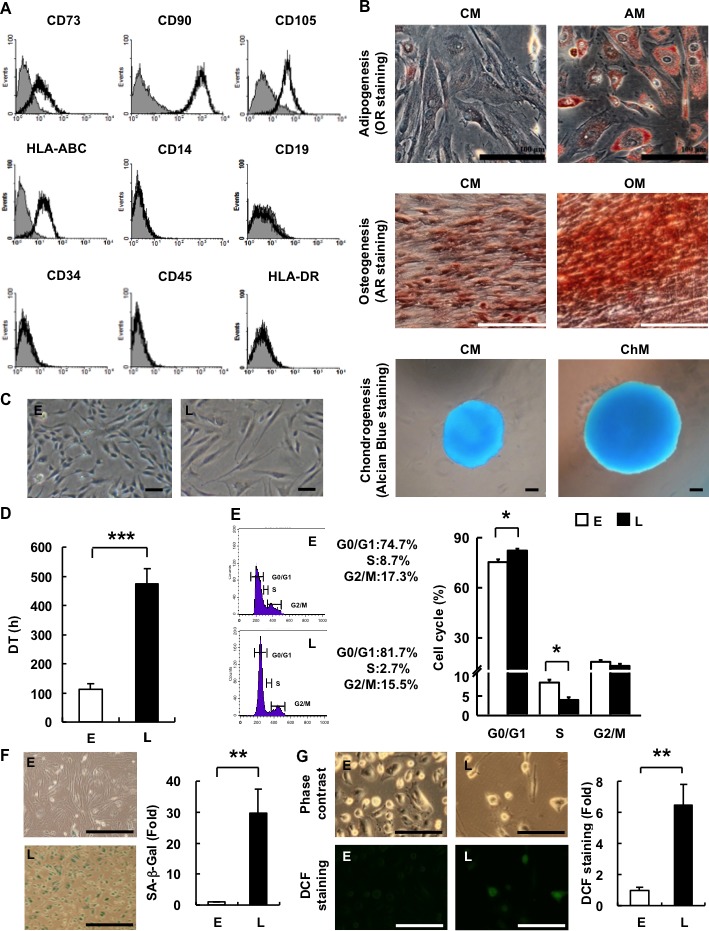
hAMSCs are multilineage cells which undergo *in vitro* replicative senescence **A.** Characterization of hAMSCs. hAMSCs were analyzed for the expression levels of CD14, CD19, CD34, CD45, CD73, CD90, CD105, HLA-ABC, and HLA-DR by FACS. **B.** Multilineage differentiation capacity of hAMSCs towards adipogenesis (AM, OR staining, magnification, ×200), osteogenesis (OM, AR staining, magnification, ×200), and chondrogenesis (ChM, Alcian Blue staining, magnification, ×200). hAMSCs cultured in CM was used as negative control. Bar = 100μm. **C.** Morphology of E- and L-hAMSCs (phase-contrast, magnification, ×200). Bar = 200μm. **D.** DT of E- and L-hAMSCs. **E.** Cell cycle analysis of E- and L-hAMSCs. **F.** SA-β-Gal staining of E- and L-hAMSCs. Bar = 500μm. Percentage of SA-β-Gal positive cells was calculated and result was shown as fold change relative to E-hAMSCs **G.** DCF staining of E- and L-hAMSCs for ROS. Bar = 200μm. Percentage of DCF positive cells was calculated and result was shown as fold change relative to E-hAMSCs. *p < 0.05; **p < 0.01; ***p < 0.001. hAMSCs, human adipose tissue-derived mesenchymal stem cells; AM, adipogenic medium; OR, Oil Red O; OM, osteogenic medium; AR, Alizarin Red; ChM, chondrogenic medium; CM, complete medium; E-hAMSCs, early-passage hAMSCs; L-hAMSCs, late-passage hAMSCs; DT, doubling times; SA-β-Gal, senescence-associated β-galactosidase; DCF, 2′, 7′-dichlorofluorescein; ROS, reactive oxygen species.

hAMSCs express the cell surface markers CD73, CD90, CD105, HLA-ABC, and lack expression of CD14, CD19, CD34, CD45, and HLA-DR, similar to BMMSCs (Figure 1A). Moreover, hAMSCs possess multi-lineage differentiation potential, including osteogenesis, adipogenesis, and chondrogenesis (Figure 1B). Every batch of culture-expanded cells (Passage 2-3) was characterized by the determination of their surface markers expression and differentiation potential before using experiments. These results confirmed that the isolated hAMSCs are multipotent MSCs [13].

### hAMSCs undergo *in vitro* replicative senescence

In the process of *in vitro* expansion to meet the demands of therapeutic use, BMMSCs undergo replicative senescence. We found that hAMSCs undergo *in vitro* replicative senescence with passaging as well. Early-passage hAMSCs (E-hAMSCs)—which we defined as having a doubling time (DT) of less than 200 hrs—were smaller and had a fibroblast-like morphology, while late-passage hAMSCs (L-hAMSCs)— with a DT of more than 400 hrs—had a flat and hypertrophic phenotype (Figure 1C, 1D). As passage number increased, proliferative capacity decreased and the expanded cell number gradually declined as the DT of E-hAMSCs, which averaged 130 hours, increased on average 3.7-fold with L-hAMSCs. We also examined for changes in the cell cycle and consistent with the above results, L-hAMSCs showed an 11% increase in the G1 phase when compared with E-hAMSCs (Figure 1E), suggesting the reduced proliferation rate is due to the decreased S phase and increased G1 checkpoint control. Cellular senescence is not only associated with increased cell volume and decreased proliferative capacity, but also expression of neutral senescence-associated β-galactosidase (SA-β-Gal) activity [14, 15], and we found that L-hAMSCs showed positive SA-β-Gal staining whereas E-hAMSCs did not (Figure 1E). Many reports have indicated that ROS accumulate in senescence cells [16]. We therefore analyzed for intracellular ROS levels in hAMSCs by staining with 2′, 7′-dichlorofluorescein (DCF), which detects H_2_O_2_ and superoxide anion levels. Compared to E-hAMSCs, L-hAMSCs stained positively with DCF (Figure 1G). Collectively, these results indicate that hAMSCs undergo senescence during *in vitro* expansion.

### Replicative senescence results in increased adipogenic but decreased osteogenic potential in hAMSCs

**Figure 2:**
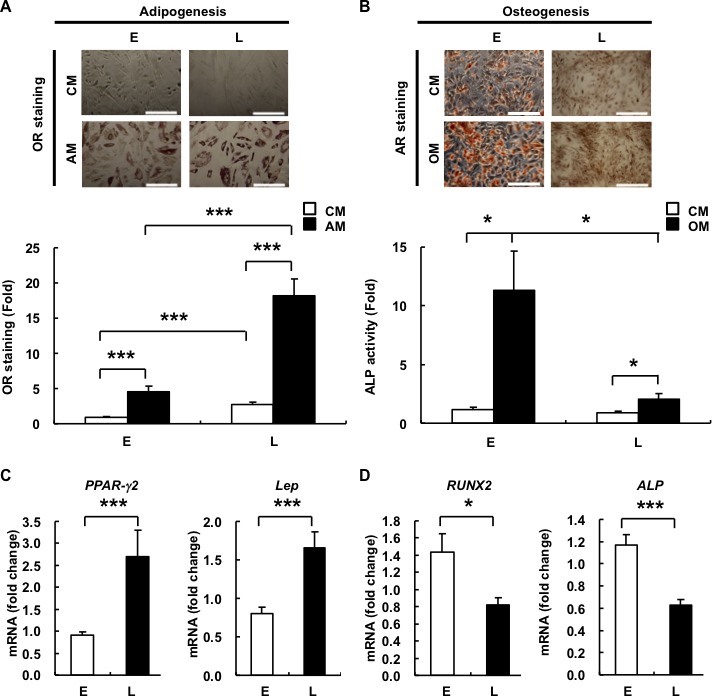
L-hAMSCs show an increased adipogenic but decreased osteogenic differentiation capacity E- and L-hAMSCs were cultured in CM, AM **A.** or OM **B.** and stained by OR staining or AR staining/ALP activity for adipogenesis and osteogenesis, respectively. Bar = 200μm. The staining intensity of 4^th^ passage cells cultured in CM was set at 1. RT-PCR was performed to detect the expression level of **C.** *PPAR-γ2*, *Lep*, **D.** *RUNX2*, and *ALP* in E- and L-hAMSCs. *β-actin* was used as internal control. Gene expression level of 4^th^ passage cells was set at 1. Data are representative of 5 independent experiments, and shown as mean values ± SEM. *p < 0.05; ***p < 0.001. L-hAMSCs, late-passage hAMSCs; E-hAMSCs, early-passage hAMSCs; CM, complete medium; AM, adipogenesis medium; OM, osteogenesis medium; OR, Oil Red O; AR, Alizarin Red; ALP, alkaline phosphates; RT-PCR, reverse transcription-polymerase chain reaction; PPAR-γ2, peroxisome proliferator-activated receptor-gamma 2; Lep, leptin; RUNX2, runt related transcription factor 2.

One of the key characteristics of any stem cell is the ability for multilineage differentiation. As such, senescence in BMMSCs has been evaluated for alterations to this capacity but the data has been mixed, with some reports showing changes in differentiation capacity with donor age and *in vitro* expansion [8, 9, 17], but not by others [7]. To answer whether senescence affects the differentiation capacity of hAMSCs, we performed adipogenic and osteogenic differentiation on E- and L-hAMSCs. We found after culturing in adipogenesis medium (AM), L-hADMCs showed an over 3-fold increase in oil droplet formation as measured with Oil Red O (OR) staining compare to E-hAMSCs. Conversely, when cultured in osteogenesis medium (OM), E-hAMSCs showed stronger Alizarin Red (AR) staining for calcium, and over 5-fold increase in alkaline phosphatase (ALP) activity—an osteogenesis marker—compare to L-hAMSCs (Figure 2A, 2B). To investigate whether senescence affects lineage commitment at a molecular level, we assessed for changes in gene expression of two major lineage-commitment TFs, peroxisome proliferator-activated receptor-gamma 2 (PPAR-γ2) and runt related transcription factor 2 (RUNX2), for adipogenic and osteogenic commitment, respectively. Consistently, we found that in L-hAMSCs compared to E-hAMSCs, *PPAR-γ2* expression was increased while *RUNX2* expression was decreased, respectively (Figure 2C, 2D). In addition, we also detected the expression of *leptin* (*Lep*) and alkaline phosphatase (*ALP*), which are adipogenic and osteogenic markers, respectively, and expression levels of these downstream lineage markers correlate with their respective master TFs. We also assessed whether chondrogenic differentiation was affected by assessing for expression levels changes in Sox9, the master chondrogenic TF, but found no significant difference between E- and L-hAMSCs (data not shown). Taken together, our data show that senescence shifts the differentiation potential from osteogenesis to adipogenesis in hAMSCs.

### Upregulated antioxidative as well as cell-cycle arrest genes, and downregulated pluripotency genes are seen in senescent hAMSCs

**Figure 3:**
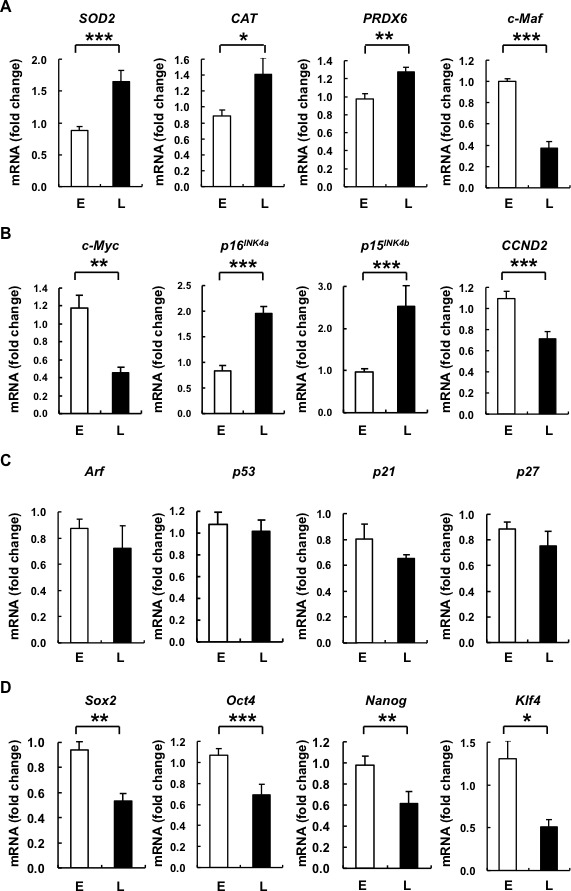
L-hAMSCs show transcriptional increases in expression of antioxidant enzymes, cell cycle arrest genes p16^*INK4a*^ and p15^*INK4b*^, and decreased expression of *c-Maf* and pluripotency genes RT-PCR was performed to detect the expression level of **A.** anti-ROS-associated genes (*SOD2*, *CAT*, *PRDX6*, and *c-Maf*), **B.** and **C.** cell cycle-associated proteins (*c-Myc*, *p16^INK4a^*, *p15^INK4b^*, *CCND2*, *Arf*, *p53*, *p21*, and *p27*), and **D.** pluripotency markers (*Sox2*, *Oct4*, *Nanog*, and *Klf4*) in E- and L-hAMSCs. *β-actin* was used as internal control. The gene expression level of 4^th^ passage cells is set at 1. Data are representative of 5 independent experiments, and shown as mean values ± SEM. *p < 0.05; **p < 0.01; ***p < 0.001. L-hAMSCs, late-passage hAMSCs; RT-PCR, reverse transcription-polymerase chain reaction; SOD2, superoxide dismutases 2; CAT, catalase; PRDX6, peroxiredoxin 6; CCND2, cyclin D2; E-hAMSCs, early-passage hAMSCs.

To further elucidate the molecular pathways involved in hAMSC senescent changes resulting in elevated ROS, decreased proliferative capacity, and altered differentiation capacity, we assayed for changes in the expression of related genes in E- and L-hAMSCs. We found that *superoxide dismutases 2* (*SOD2*), *catalase* (*CAT*), and *peroxiredoxin 6* (*PRDX6*) levels were significantly increased in L-compared to E-hAMSCs; levels of *c-Maf*—a TF known to be important in immune cell development [18] and recently found to mediate age- and ROS-related MSC differentiation capacity [6, 19]—were decreased in L-hAMSCs (Figure 3A). The growth arrest so prominent in most senescent cells is known to involve the Arf-p53-p21 and/or p15^INK4b^ /p16^INK4a^ –pRb pathways. In senescence, these pathways are activated and result in G1-cell cycle arrest [20]. We therefore assayed for changes in expression of these cell cycle-associated genes in E- and L-hAMSCs. We found that *p15^INK4b^* and *p16^INK4a^*, but not *Arf*, *p53*, *p21* or *p27* were significantly increased in L-compared to E-hAMSCs. In addition, the expression of *cyclin D2* (*CCND2*), a G1-cell cycle regulatory gene, was decreased in L-hAMSCs. (Figure 3B, 3C). Interestingly, *c-Myc*, a TF now known to be important in both cell proliferation and inducing pluripotency [21], was also significantly downregulated in L-hAMSCs as well. Furthermore, we found that the embryonic stem cell pluripotency genes *Sox2*, *Oct4*, and *Nanog* [22] were significantly decreased in L- compared to E-hAMSCs. *Klf4*, another gene found to be important in maintaining/inducing pluripotency [21, 23] was significantly downregulated with hAMSC senescence as well (Figure 3D). These results suggest that senescence in hAMSCs involve induction of genes for antioxidant enzymes and p15^INK4b^ /p16^INK4a^, as well as downregulation of *c-Maf* and pluripotency genes.

### Exogenous ROS (H_2_O_2_) inhibits proliferation and results in differentiation bias towards adipogenesis in E-hAMSCs

**Figure 4:**
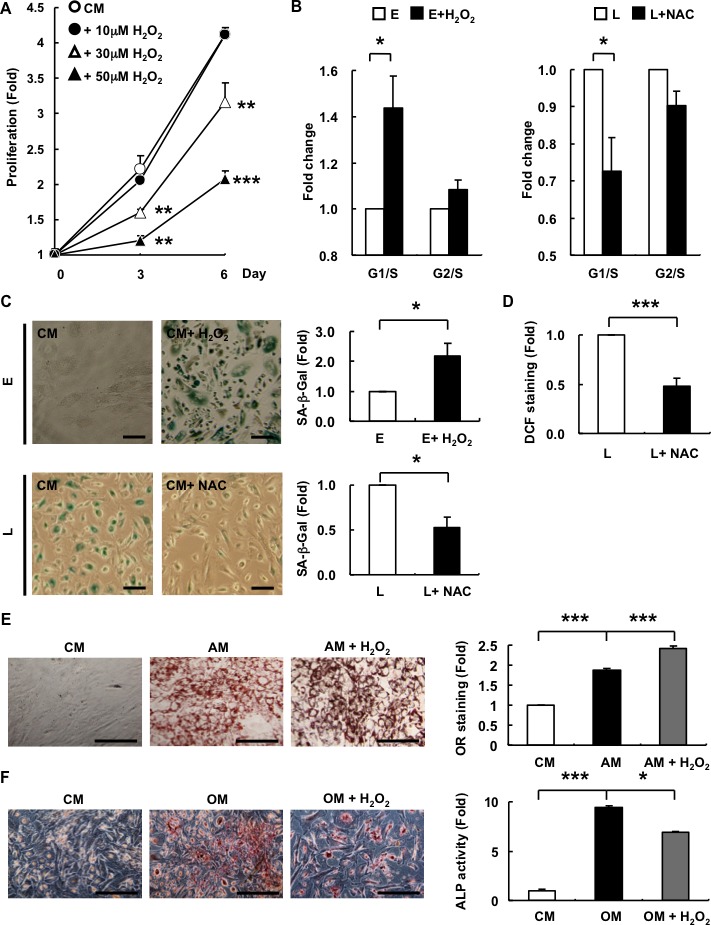
Exogenous ROS decreases the proliferation potential and alters differentiation capacity from osteogenesis towards adipogenesis in E-hAMSCs **A.** *In vitro* proliferation of E-hAMSCs cultured in CM with or without H_2_O_2_ treatment. **B.** The measurement of cell cycle distribution was described in “Materials and Methods”. E-hAMSCs were treated with or without H_2_O_2_ (50μM, 24 h), and L-hAMSCs were treated with or without NAC (50μM, 24 h) **C.** SA-β-Gal staining and quantification were described in “Materials and Methods”. E-hAMSCs were treated with or without H_2_O_2_ (50μM, 12 days), and L-hAMSCs were treated with or without NAC (50μM, 6 h). Bar = 200μm **D.** DCF staining and quantification were described in “Materials and Methods”. L-hAMSCs were treated with or without NAC (50μM, 6 h). **E.** Adipogenesis and **F.** osteogenesis of E-hAMSCs cultured in CM, AM or OM with or without H_2_O_2_ treatment (30μM), respectively. Cells were cultured 2 days for adipogenesis and 21 days for osteogenesis. Bar = 200μm. The staining intensity of E-hAMSCs cultured in CM was set at 1. All data are representative of at least three independent experiments, and shown as mean values ± SEM. *p < 0.05; **p < 0.01; ***p < 0.001. ROS, reactive oxygen species; E-hAMSCs, early-passage hAMSCs; CM, complete medium; H_2_O_2_, hydrogen peroxide; SA-β-Gal, senescence-associated β-galactosidase; DCF, 2′, 7′-dichlorofluorescein; L-hAMSCs, late-passage hAMSCs; H_2_O_2_, hydrogen peroxide; NAC, *N*-acetyl cysteine.

H_2_O_2_ induces either apoptosis or cellular senescence in cultured cells [24]. We found that levels of ROS are increased in hAMSCs with senescence (Figure 1G) and to further elucidate the mechanism involved, we first verified whether exogenous H_2_O_2_ would trigger cellular senescence in hAMSCs by assessing for various senescence-related parameters, including cell proliferation, cell cycle dynamics, and SA-β-Gal staining, as well as functional assays of multilineage differentiation capacity. We found that sublethal doses of H_2_O_2_ can induce inhibition of proliferation and G1/S cell cycle arrest in E-hAMSCs (Figure 4A, 4B). Treatment of sublethal doses of H_2_O_2_ to E-hAMSCs also resulted in a flattened cell morphology and an enlarged cell size (data not shown). Moreover, the senescence of L-hMSCs could be rescued by *N*-acetyl cysteine (NAC), a H_2_O_2_ scavenger, as revealed by reversal of cell cycle senescent-changes (Figure 4B), SA-β-Gal staining (Figure 4C), as well as DCF staining (Figure 4D). To ascertain whether alteration of differentiation capacity in senescent hAMSCs (Figure 2) was mediated by ROS, we simultaneously treated E-hAMSCs with H_2_O_2_ while performing adipogenic or osteogenic differentiation. We found that, compared to untreated cells, adipogenesis is further enhanced in H_2_O_2_-treated E-hAMSCs cultured in AM (Figure 4E). Conversely, H_2_O_2_ decreased osteogenesis of E-hAMSCs cultured in OM (Figure 4F). These finding reveal that ROS play a role in mediating the functional changes in hAMSCs brought about by senescence.

### c-Maf is involved in H_2_O_2_-mediated reduced pluripotency and proliferation as well as differentiation bias changes in hAMSCs

**Figure 5:**
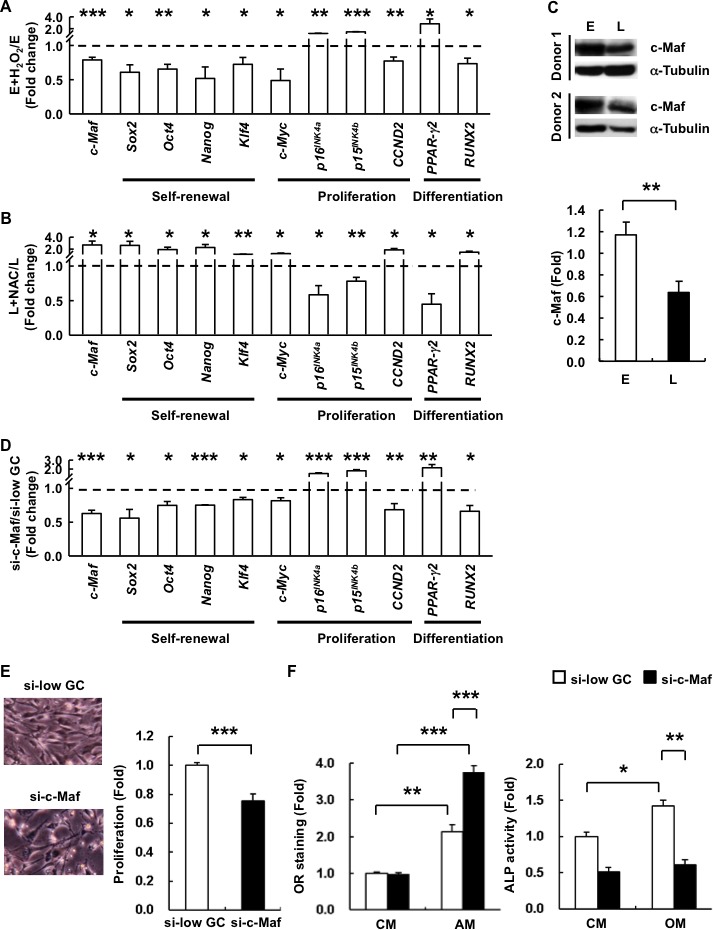
c-Maf is involved in H_2_O_2_-induced replicative senescence and adipogenic differentiation bias in hAMSCs RT-PCR was performed to detect the expression of senescence-related genes, including differentiation-associated genes, cell cycle-associated proteins, and pluripotency markers in **A.** E- and **B.** L-hAMSCs treated with or without H_2_O_2_ or NAC, respectively. The expression of each gene was normalized against β-actin and the result was presented as fold change compared with the untreated cells **C.** The expression of c-Maf in E- and L-hAMSCs was detected by western blotting. α-Tubulin was used as internal control. **D.** RT-PCR was performed to detect the expression of senescence-related genes in E-hAMSCs after RNA silencing of *c-Maf* by siRNA specific for c-Maf (si-c-Maf) or non-target siRNA (si-low GC). The expression of each gene was normalized against β-actin and the result was presented as fold change compared with si-low GC-treated cells. **E.** Morphology and *in vitro* proliferation of si-c-Maf- and si-low GC-E-hAMSCs. **F.** si-c-Maf- and si-low GC-E-hAMSCs were cultured in CM, AM or OM and measured by OR staining or ALP activity for adipogenesis and osteogenesis, respectively. The staining intensity of si-low GC-E-hAMSCs in CM was set at 1. All data presented are mean values ± SEM from at least three independent experiments. *p < 0.05; **p < 0.01; ***p < 0.001. H_2_O_2_, hydrogen peroxide; hAMSCs, human adipose tissue-derived MSCs; RT-PCR, reverse transcription-polymerase chain reaction; E-hAMSCs, early-passage hAMSCs; L-hAMSCs, late-passage hAMSCs; H_2_O_2_, hydrogen peroxide; NAC, *N*-acetyl cysteine; siRNA, small interfering RNA; si-c-Maf, specific knockdown of c-Maf; CM, complete medium; AM, adipogenesis medium; OM, osteogenesis medium; OR, Oil Red O; ALP, alkaline phosphatase.

To elucidate the molecular mechanisms by which ROS-induced senescence mediate changes to cell proliferation and lineage commitment in hAMSCs, we treated E-hAMSCs with H_2_O_2_ or L-hAMSCs with NAC and assessed for changes in gene expression of lineage master TFs and senescence-related genes. Indeed, we found that H_2_O_2_ reduces the genetic programs of self-renewal, cell cycle, and osteogenic differentiation at the transcriptional level in E-hAMSCs (Figure 5A). In L-hAMSCs, the expression of these genes could be enhanced by NAC (Figure 5B). We then focused on c-Maf, which has been recently been found to participate in age- and ROS-related MSC differentiation capacity changes [6, 19]. We found that *c-Maf* expression is decreased by addition of H_2_O_2_ in E-hAMSCs and restored by NAC-treated L-hAMSCs (Figure 5A, 5B). Moreover, expression of c-Maf was downregulated in L-hAMSCs (Figure 3A) in line with the protein levels (Figure 5C). To ascertain the functional role of c-Maf in hAMSC senescent processes, we performed *c-Maf* knockdown experiments in E-hAMSCs by small interfering RNA (siRNA). Specific knockdown of *c-Maf* (si-c-Maf) in hAMSCs effectively decreased *c-Maf* expression (Figure 5D), and we found that the expression of self-renewal-, proliferation-related genes and *RUNX2* was decreased; however, *p16^INK4a^*, *p15^INK4b^*, and *PPAR-γ2* expression was increased in si-c-Maf-hAMSCs. Furthermore, si-c-Maf-hAMSCs had a flat and hypertrophic phenotype, *in vitro* proliferative capacity (Figure 5E), as well as a differentiation bias towards adipogenesis and away from osteogenesis (Figure 5F). These results demonstrate that c-Maf is involved in both the proliferative and differentiation capacity changes brought about by ROS-induced senescence in hAMSCs.

### c-Maf binds directly to senescence-altered genes which can be reduced by exogenous H_2_O_2_

**Figure 6:**
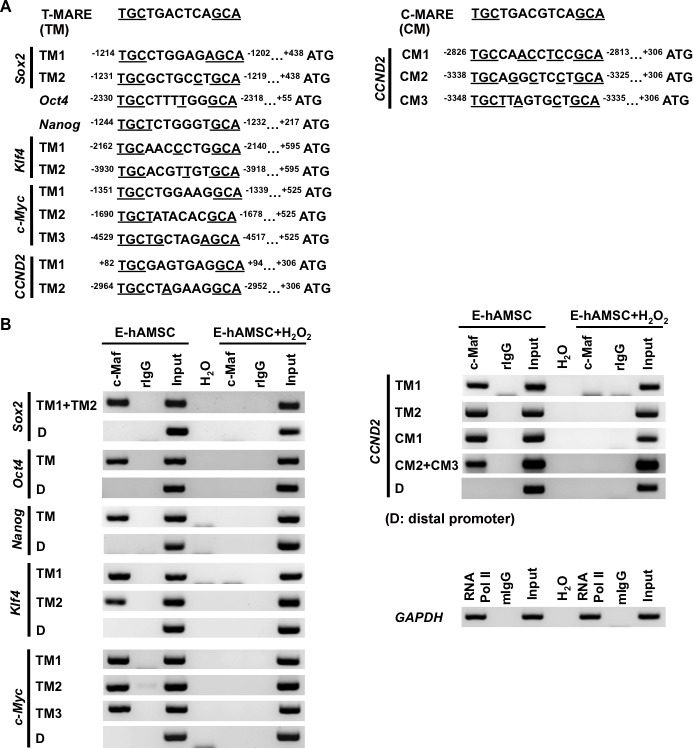
c-Maf directly binds to senescence-altered genes in hAMSCs which can be reduced by disrupted with exogenous H_2_O_2_ **A.** Schematic representation of the location of each palindromic c-Maf-responsive element on senescence-altered gene promoters aligned with Maf recognition element (T- and C-MARE) consensus sequences. Sequences that are identical to those in MARE are underlined. The numbers of the MARE site indicated are relative to the transcription start site (+1). Translation initiation site, ATG; T-MARE, TM; C-MARE, CM. **B.** ChIP analysis of senescence-altered gene promoters in E-hAMSCs treated with or without H_2_O_2_. Input, cell lysates (1%; for assessment without prior immunoprecipitation); D, distal promoter; c-Maf, anti-c-Maf; rIgG, anti-rabbit IgG; RNA Pol II, anti-RNA Polymeras II; mIgG, anti-mouse IgG; H_2_O, negative control for PCR assay. hAMSCs, human adipose tissue-derived MSCs; H_2_O_2_, hydrogen peroxide; T-MARE, phorbol 12-*O*-tetradecanoate-13-acetate-responsive element; C-MARE, cyclic AMP-responsive element; MARE, Maf recognition element; ChIP, chromatin immunoprecipitation; E-hAMSCs, early-passage hAMSCs.

To elucidate whether c-Maf mediate senescence changes in hAMSCs by directly binding to the promoter regions of senescence-altered genes, we performed sequence analysis in these regions to identify *cis*-acting regulatory Maf recognition element (MARE), the binding element for c-Maf [25]. Currently, known palindromic MAREs include the phorbol 12-*O*-tetradecanoate-13-acetate-responsive element (T-MARE; TGCTGACTCAGCA) and the cyclic AMP-responsive element (C-MARE; TGCTGACGTCAGCA) [26]. The TGC/GCA flanking motif is crucial for Maf binding and is conserved. Using *in silico* analysis, we identified various putative T- and C-MARE sites on the promoter regions of senescence-altered genes in position from 5000 base pair (bp) upstream to translation initiation site (Figure 6A). Software analysis revealed that nearly all predicted MARE sites were at distal promoter regions (more than 1000 bp from the transcription start site); for T-MARE, elements could be found on the promoters of *Sox2*, *Oct4*, *Nanog*, *Klf4*, *c-Myc*, and *CCND2,* whereas for C-MARE, sites only could be found on *CCND2* (Figure 6A). To investigate whether c-Maf directly interacts with these MARE sites and the putative relationship with H_2_O_2_ exposure, a chromatin immunoprecipitation (ChIP) assay was performed using either untreated or H_2_O_2_-treated E-hAMSCs. We found that c-Maf directly bound to T-MARE sites on the promoters of *Sox2*, *Oct4*, *Nanog*, *Klf4*, *c-Myc*, and *CCND2*; c-Maf binding could be demonstrated in C-MARE sites on the promoters of *CCND2* (Figure 6B). Moreover, the binding capacity of c-Maf at all sites was strongly decreased by exogenous H_2_O_2_. Taken together, the data demonstrate a mechanism by which ROS through c-Maf alters differentiation as well as proliferative/self-renewal capacity, the latter via transcriptional regulation of cell cycle- and pluripotency-related genes into a cellular program of senescence in hAMSCs.

## DISCUSSION

**Figure 7:**
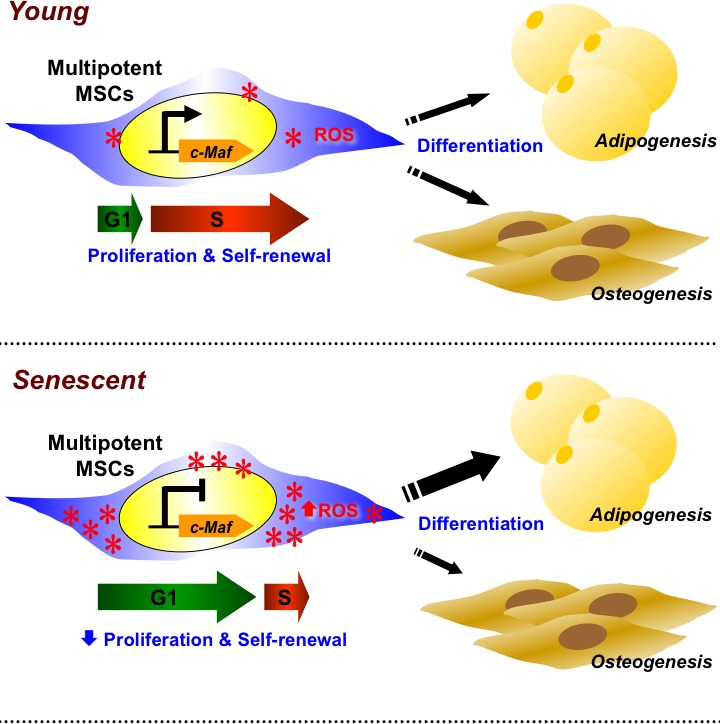
c-Maf directly regulates ROS-mediated the senescent changes of proliferation, self-renewal, and differentiation capacity in hAMSCs Senescent L-hAMSCs and H_2_O_2_-treated E-hAMSCs undergo ROS-mediated proliferative decline, cell cycle arrest in G0/G1, diminished expression of pluripotency factors, and differentiation bias towards adipogenesis and away from osteogenesis. These molecular changes which then affect multiple stem cell functions are directly mediated by c-Maf. ROS, reactive oxygen species; hAMSCs, human adipose tissue-derived MSCs; L-hAMSCs, late-passage hAMSCs; H_2_O_2_, hydrogen peroxide; E-hAMSCs, early-passage hAMSCs.

MSCs are a highly relevant stem cell population for clinical applications due to their multilineage differentiation potential, immunomodulatory effects, and ability to home to sites of inflammation [27, 28]. However, while many clinical trials currently use MSCs, the rarity of these cells—regardless of source—require vigorous *ex vivo* expansion prior to clinical use. Such expansion will cause replicative senescence which limits numbers of MSCs for utilization or adversely alter functional capacity and therapeutic effects. Thus, understanding the mechanisms involved in MSC replicative senescence can have therapeutic relevance, as well as elucidate the biology of MSC self-renewal. We found that hAMSCs, a popular source of MSCs isolated from adipose tissue, undergo replicative senescence in which ROS play a role (Figure 7). Both L-hAMSCs and H_2_O_2_-treated E-hAMSCs showed reduced population doubling level, cell cycle arrest at G1 phase, alteration of differentiation capacity from osteogenesis towards adipogenesis, and—on a molecular level—increased expression of antioxidant enzymes as well as p15^INK4b^and p16^INK4a^ senescence-related genes but decreased expression of pluripotency factors and c-Maf. These multitudes of changes in the senescent process of hAMSCs appear to be directly mediated by c-Maf. Our findings offer a molecular explanation for the effect of ROS on altering both the proliferative and differentiation capacity involved in replicative senescence of hAMSCs.

c-Maf is a large Maf TF[12] and involves in a number of developmental processes, including immune cellular differentiation [18, 29] and ocular lens development [30, 31]. In addition, reduction of c-Maf has been reported to be associated with a reduced osteogenic capacity in aged MSCs [19]. This study also showed the involvement of ROS and found that the osteogenic effect of c-Maf as due to its role as a co-factor of RUNX2, the master TF for osteogenesis. Subsequently, we showed that accumulation of the ROS in human fetal MSCs affects lineage commitment by decreasing c-Maf expression [6]. However, whether c-Maf is also involved other aspects of MSC senescence—especially the profound decrease in proliferative capacity—has not been evaluated. We demonstrate in this report that c-Maf directly regulates the transcriptional expression of major programs of cell proliferation, including *CCND2* and various pluripotency factors; moreover, binding of c-Maf to the promoter regions of these pluripotency and cell cycle-related genes was disrupted by addition of exogenous ROS. This latter finding is not only strongly supported by the known link of oxidative stress to cellular senescence, but also by the increasing reports of the protective effects of hypoxic culture on MSCs [32].

We found that MARE can be found on the promoter region of many pluripotency factors. While these factors are best associated with pluripotent stem cells such as embryonic stem cells (ES cells) and induced pluripotent stem cells (iPSCs), it has been increasingly reported that some of these factors may also be important in MSC biology [33, 34]. Indeed, a few recent reports show that decline in self-renewal pathways may be one of the major mechanisms attributing to MSC senescence [35, 36]; however, the details involved regarding transcriptional silencing of pluripotency genes in senescent MSCs are poorly understood. Indeed, one report has shown that hypoxia enhances the efficiency in the generation of iPSCs [37] but mechanisms involved were not examined. Given that our findings on the adverse ROS effect has on expression of pluripotency factors and cell proliferation, it is tempting to speculate on the role that c-Maf may play in the iPSC reprogramming process.

The p16 ^INK4a^-p15^Ink4b^/Rb1 and ARF/p21/p53 cell cycle inhibitory pathways represent two important pathways controlling proliferation, and their inactivation can extend the limited division number of mitotic cells in culture [38]. Our data supports the p15^Ink4b^and p16^Ink4a^ as the major regulators inducing senescence in hAMSCs. p15^Ink4b^ and p16^Ink4a^ are considered to be robust biomarkers for cellular senescence, and at the forefront of cell cycle inhibition as it binds specifically to the CDKs, displacing cyclin-D and thereby arresting cells in G1 phase. We found that as hAMSCs undergo senescence, an increased number of cells in G1 phase arrest were seen (Figure 1E), with concomitant increases in the expression of *p16 ^INK4a^* and *p15^Ink4b^*, along with decreased *CCND2* expression (Figure 3B). Interestingly, *p15^INK4b^*, *p16^INK4a^*, and *ARF* are encoded by a single locus; however, c-Maf specifically affects *p16^INK4a^* and *p15^INK4b^*, but not *ARF*. It may be that the p16^INK4a^ pathway is of particular importance in the senescence of stem cells [39, 40]. Along with these reports, our data demonstrate that the p16^INK4a^ pathway may be especially relevant to stem cell senescence, and how ROS may influence this pathway through c-Maf.

In conclusion, we provide mechanistic insight into the molecular changes of ROS-mediated senescent changes in hAMSCs. We found that c-Maf is transcriptional regulated by ROS and acts as a master TF in controlling multiple stem cell programs of proliferation, self-renewal, and lineage commitment. Our findings demonstrate the strong detrimental effects of ROS on multiple aspects of stem cell function with c-Maf playing a central role in these processes.

## MATERIALS AND METHODS

### Isolation and cell culture of hAMSCs

Adipose tissue from healthy donors was obtained with informed consent approved by the institutional review board. hAMSCs were isolated and expanded as previously described [41]. Briefly, the tissues were first washed at least three times with PBS to remove blood. Tissues were then digested with Collagenase Type I (Invitrogen, Carlsbad, CA, USA) for 1 h at 37°C, and centrifuged to obtain single-cell suspension. Cells were cultured in low glucose-DMEM (Invitrogen) supplemented with 10% FBS (HyClone, Logan, UT, USA), 100 U/ml penicillin, 100 g/ml streptomycin (Invitrogen), and 2 mM L-glutamine (Invitrogen) at 37°C with 5% CO_2_. In some experiments, cells were treated with H_2_O_2_ (Sigma-Aldrich, St. Louis, MO, USA) and NAC (Sigma-Aldrich).

### Immunophenoyping

Cells were stained for expression of various surface markers and analyzed with a BD FACSCalibur (BD Biosciences, San Jose, CA, USA) as previously reported [42, 43]. All antibodies for flow cytometry analysis were purchased from BD Biosciences. Each analysis included the appropriate FITC- and PE-conjugated isotype controls.

### Differentiation studies

Differentiation and characterization studies were performed as previously described [42-44]. Briefly, for adipogenic differentiation, cells were cultured in adipogenesis medium (AM) composed of complete medium (CM), 0.5 μM isobutyl-methylxanthine, 1 μM dexamethasone, 10 μM insulin, and 60 μM indomethacin. Adipogenic differentiation was evaluated using Oil Red O (OR) staining as an indicator of intracellular lipid accumulation. For osteogenic differentiation, cells were cultured in osteogenesis medium (OM) composed of complete medium (CM), 0.1 μM dexamethasone, 10 mM β-glycerol phosphate, and 50 μΜ ascorbate. Alizarin Red (AR) staining and ALP activity were performed to estimate calcium deposits. For chondrogenic differentiation, cells were cultured in serum-free medium with the addition of 10 ng/ml TGF-β3 (R&D Systems Inc., Minneapolis, MN, USA). Alcian Blue staining was performed to visualize the chondrogenic differentiations. Cells cultured in complete medium (CM) represent control condition. To quantify the results of adipogenic and osteogenic differentiation, OR and AR were extracted from cells cultured in complete and differentiation medium. The absorbance of extracted dye was then measured at 520 nm by UV spectrophotometer. ALP activity also be used to quantify the results of osteogenic differentiation, it was assayed colorimetrically by incubating protein lysates with the substrate p-NPP (Sigma-Aldrich) at RT for 5hr. The absorbance was detected at OD 405 *nm* and normalized to the corresponding protein content. Experiments for the quantitative assessment of adipogenic and osteogenic differentiation were performed at least three replicates and data were presented as fold change relative to control condition. All reagents were purchased from Sigma-Aldrich.

### Cell proliferation and population DT assay

Proliferation rate was determined by counting cell number and calculating population DT as previously reported [6]. Cells were cultured 1×10^4^ cells/cm^2^ beginning from the 4^th^ passage. At sub-confluent growth at a density of 80%, cells were trypsinized and counted manually by hemocytometer. The DT (hours required for the cell number to double) is an index reflecting the growth of cultured cells, and an increase of DT reflects a deceleration in cell growth. At each passage, DT was determined by the formula: (T)×log(2)/log(N2/N1). T is the time between periods. N1 and N2 is the number of cells at confluence and initial cell number, respectively.

### Cell cycle analysis

Cell cycle analysis was performed as previously described [6]. Briefly, cells were fixed and stained with propidium iodide (PI) staining buffer (20 μg/ml PI, 0.1 mg/ml RNase A, and 1% Triton X-100; all from Sigma-Aldrich) and analyzed with a BD FACSCalibur using MoDfit LT (BD Biosciences).

### SA-β-Gal staining

β-Gal activity of senescent cells was detected by senescence SA-β-Gal staining kit (Cell Signaling, Danvers, MA, USA) as previously described [6]. SA-β-Gal positive cells were shown as blue-green color and observed by a microscope (200 x magnifications). The percentage of the blue-green stained cells was counted in three random fields for each triplicate sample. hAMSCs were determined as senescent if the morphology was flattened and widened, 2-fold change in DT, and when >70% of the cells were β-Gal positive cells, whereas pre-senescent cells contained <10% β-Gal positive [14].

### Measurement of intracellular ROS levels

Intracellular ROS levels were measured using the cell permeable substrate, 2′, 7′-dichlorodihydrofluorescein diacetate (H_2_DCF-DA; Sigma-Aldrich) as previously described [6]. Intracellular ROS was detected by H_2_DCF-DA staining. Images were obtained by a fluorescent microscope (200 × magnifications) and the number of fluorescent positive cells was counted randomly in three fields for each triplicate sample. Percentage of fluorescent positive cells was calculated and result was shown as fold change relative to control cells.

### RNA isolation and reverse transcription-polymerase chain reaction (RT-PCR)

Total RNA was extracted using Trizol reagent (Invitrogen) and RT-PCR was performed as previously reported [6]. Primers used in the experiment were listed in Supplementary Table S1. All primer sequences were generated from established GenBank sequences.

### Western blot analysis

Protein was obtained from cells as previously reported [45]. Primary antibodies against c-Maf and α-Tubulin were obtained from Santa Cruz Biotechnology (Santa Cruz Biotechnology, Dallas, TX, USA).

### RNA silencing

RNA silencing of *c-Maf* in hAMSCs was performed by using siRNA targeting *c-Maf* (Stealth Select RNAi™ siRNA, Invitrogen) along with low GC siRNA (Stealth^TM^ negative control) according to manusfacturer's recommendation. siRNAs were transfected into cells by using Lipofectamine™ RNAiMAX (Invitrogen) following the manufacturer's recommended procedures. Briefly, the transfection mixture was added to the cells 24 hours after plating in serum-free medium for 4 to 6 hours, and replaced with CM. After 3 days of culture, knockdown of *c-Maf* in hAMSCs was confirmed by RT-PCR.

### ChIP assay

ChIP was carried out using EZ-Zyme Chromatin Prep kit (Millipore, Billerica, MA, USA) and Magna ChIP A/G kit (Millipore) as previously reported [46]. Briefly, chromatin-protein complex was prepared according to the manufacturer's recommendations. The complex was then immunoprecipitated using polyclonal antibody against c-Maf (Santa Cruz Biotechnology) or RNA Pol II or isotopes (rIgG or mIgG) antibody or without antibody. PCR was performed to monitor the amount of c-Maf bound to the MARE site of promoters with specific primers listed in Supplementary Table S2. The PCR cycles were as follows: 94°C for 10 min, one cycle; 94°C for 30 seconds, 60°C for 30 seconds and 72°C for 30 seconds, 35 cycles; and 72°C for 10 min, one cycle. The PCR products were then analyzed in 2% agarose gels.

### Statistical analysis

Analysis of statistics was carried out using GraphPad Prism 5.0 software (GraphPad Software Inc., La Jolla, CA, USA). All data are presented as mean ± SEM, and *p* < 0.05 was considered statistically significant. All experiments were performed at least in triplicate.

## SUPPLEMENTARY MATERIAL TABLES


