# Supplementary material for: c-Maf regulates pluripotency genes, proliferation/self-renewal, and lineage commitment in ROS-mediated senescence of human mesenchymal stem cells
Source: Oncotarget. 2015 Oct 19;6(34):35404–18. doi: 10.18632/oncotarget.6178 (PMC4742114; doi:10.18632/oncotarget.6178)
Supplement: Supplementary file 1 [file oncotarget-06-35404-s001.pdf]

c-Maf regulates pluripotency genes, proliferation/self-renewal, and lineage commitment in ROS-mediated senescence of human mesenchymal stem cells

Supplementary Material

Supplementary Table S1: Lists of primers used for RT-PCR assay.
Supplementary Table S2: Lists of primers used for ChIP-PCR assay.

Table S1. Lists of Primers Used for RT-PCR Assay

Gene	Forward Primer	Reverse Primer
PPAR-γ2	CGATGGGGATGCTCATAA	CTTTTGGCATACTCTGTGAT
Lep	TTCACACACGCAGTCAGT CTCC	GGCATACTGGTGAGGATCTG
RUNX2	ACCCAGAAGGCACAGACAG	TGGTAGAGTGGATGGACGG
ALP	AGCTGAACAGGAACAACGTGA	CTTCATGGTGCCCGTGGTC
SOD2	TGGACAAACCTCAGCCCTAAC	AAACCAAGCCAACCCCAACC
CAT	TACTCAGGTGCGGGCATTC	TGATGAGCGGGTTACACGG
PRDX6	ACCTCCGCTACACACCCC	AGAGTGCTGATGTATGAATGG
c-Maf	GACGACCGCTTCTCCGAC	CGCTGCTCACCAACTTCTC
c-Myc	TGCCTCAAATTGGACTTTGG	GATTGAAATTCTGTGTAACTGC
$p16^{INK4a}$	TGCCTTCCCCCACTACCG	AACCCCCCTGAGCTTCCC
$p15^{INK4b}$	CGTTAAGTTTACGGCCAACG	GGTGAGAGTGGCAGGGTCT
CCND2	ATTGGCATGTCTGGTTCACA	GAACGCCAGATACCAGAAGC
Arf	TCTTGGTGACCCTCCGGAT	GGCAGTTGTGGCCCTGTAG
p53	TGTGGGGAGGAGGATGGG	AGGGAGAGGGAACAAGCAC
p21	ATGGGGCTGGGAGTAGTTG	AGACACAGAACAGTACAGGG
p27	TTGTTAGATAGCTGCATGTGG	TCAAAACTCCCAAGCACCTC
Sox2	TACCTCTTCCTCCCACTCCAT	GGTAGTGCTGGGACATGTGA
Oct4	GTGGAGAGCAACTCCGATG	TGCAGAGCTTTGATGTCCTG
Nanog	GCTTGCCTTGCTTTGAAGCA	TTCTTGACCGGGACCTTGTC
Klf4	ACGATCGTGGCCCCGGAAAAGGACC	TGATTGTAGTGCTTTCTGGCTGGGCTC
β-actin	GGCACCCAGCACAATGAAG	TGCGGTGGACGATGGAGG

Table S2. Lists of Primers Used for ChIP-PCR Assay

Genomic region	Forward Primer	Reverse Primer
Sox2: TM1+TM2	CCATCTCATCCTCCTCTAAC	CAAAGGGGCTCAAGGCCTG
Sox2: D	AAGAACAGAGCAAGTTACGTG	CTCTCCTCTCTTACCTTACAG
Oct4: TM	CCTCCTGTTCCGAAGCATG	TTCAATACCTGCCACAGGTC
Oct4: D	TGGGACCAGTTCTGATGACTC	CTGTTATGCCTGAAGGGTAG
Nanog: TM	TCGAGTCTTTGCATTGTGAAC	CTCTTAGCAAATCCCGATTATG
Nanog: D	GCCTGGCCAAAGGGTAGG	ACCAAAATGAAGATGGGAGAC
Klf4: TM1	GGTTGAACTCCTCGAGGTC	CAGAGTCGGACGCCCTTG
Klf4: TM2	ATGGGTGCAGCACACCAAC	CCTCATCTCCTTGTGAACTC
Klf4: D	CCTAACCTCTAAAGTCTACAC	ACTGGATCCCTTCCTTACAC
c-Myc: TM1	GTGCACTTTCACTAGTATTCAG	TCGATTCTGATCAAAGAAGAGGAG
c-Myc: TM2	AACACTTGAACGCTGAGCTG	CTCCACCACCTCCAAAAGAG
c-Myc: TM3	AGAAGCCCAACCCACTCAG	CATCTTTGGTCTTTCACCTC
c-Myc: D	CTACAAGCATAAGCCACCAC	GAATAGACATTTGCTGGGTTG
CCND2: TM1	CCAGCCAGCTTGCGTCAC	AGAAGGGAGGCGAGGATTG
CCND2: TM2	ACTTCAAAGCCAGAGCACAG	AAATGCACTCCACCTAGCAC
CCND2: CM1	TTTTAACACATGGCTGGAGTG	GCTCGCCATCGAATCCAATC
CCND2: CM2+CM3	TGAGCCGACCGCGGAATC	GTGTACAAAGCCTGGAGGTG
CCND2: D	TTCACGACTCTCAAGTGGAG	ATTTCTTTAGGTTATGGTCCTG
